# Persistence of resistance: a panel data analysis of the effect of antibiotic usage on the prevalence of resistance

**DOI:** 10.1038/s41429-023-00601-6

**Published:** 2023-02-28

**Authors:** Sakib Rahman, Aaron S. Kesselheim, Aidan Hollis

**Affiliations:** 1 Department of Economics, University of Calgary, Calgary, AB Canada; 2 Program on Regulation, Therapeutics, and Law, Division of Pharmacoepidemiology and Pharmacoeconomics, Department of Medicine, Brigham and Women’s Hospital and Harvard Medical School, Boston, MA USA

**Keywords:** Infectious diseases, Business and industry

## Abstract

The use of antibiotics promotes the emergence of resistant bacteria in the patient and the environment. The extent of this well-documented biological relationship is, however, not well characterized at an ecological level. To make good policy around antibiotic use, it is important to understand the empirical connection between usage and resistance. We provide a consistent approach to estimate this relationship using national-level surveillance data. This paper estimates the effect of antibiotic usage on antibiotic resistance using an 11-year panel of data on both usage and resistance for 26 antibiotic–bacteria combinations in 26 European countries. Using distributed–lag models and event-study specifications, we provide estimates of the rate at which increases in antibiotic usage at the national level affect antibiotic resistance nationally and internationally. We also calculate the persistence of resistance and analyze how resistance behaves asymmetrically with respect to increases and decreases in usage. Our analysis finds the prevalence of resistant bacteria increases immediately after usage and continues to increase for at least 4 years after usage. We show that a decrease in usage has little identifiable impact on resistance over the same period. Usage in neighboring countries increases resistance in a country, independent of usage in that country. Trends in usage-related resistance vary across European regions and across bacterial classifications.

## Introduction

Antibiotic resistance is a global crisis and was responsible for an estimated 1.27 million deaths in 2019 [1]. The potential cost to the global economy is alarming, predicted at a loss of USD100 trillion by 2050 [2]. Through natural selection, bacteria develop resistance to antibiotics, a process that has been described since the first antibiotics were discovered [3]. The extensive and injudicious use of antibiotics in humans, animals, and agriculture is believed to have induced a crisis by increasing the prevalence of resistant pathogens [4]. However, little is known about the long-term effect of antibiotic usage on the prevalence of resistance. Specifically, the extent to which antibiotic usage causes resistance at an ecological level, the persistence of resistance, and the ability to control the growth of resistance through stricter stewardship of antibiotics, are poorly understood.

It is important to understand this relationship since policies around stewardship, pricing, and investing in new antibiotic development all depend on the evolution of resistance to our existing stock of antibiotics. Researchers have applied a variety of tools to estimate the causal relationship between usage and resistance at the ecological level. Numerous cross-sectional studies have focused on the relationship between use and resistance at a national level [5–9]. Given the variation between countries, these studies can say little about the causal effect of usage on resistance. Other researchers have focused on one country, using variation over time [10, 11]. After adjusting for seasonality, such studies have shown that changes in usage are followed rapidly by corresponding changes in resistance locally, but were not designed to assess long-term effects in the wider environment. In addition, only a limited number of studies analyze the effect of a decrease in usage [12, 13]. These studies predominantly focus on specific settings, such as primary care within a specific country, and for only one bacterial pathogen. Analyzing how resistance responds to decreases in antibiotic usage is important for policy-making around stewardship. Moreover, since resistant bacteria do not respect borders, the use of antibiotics in any country has the potential to select resistant bacteria elsewhere, as colistin resistance has illustrated [14]. The international mobility of people, animals, and goods promotes the spread of antibiotic-resistant bacteria across borders [15]. The average rate at which resistance spreads across international borders has not previously been characterized even though there exist theoretical work and descriptive studies [16–18].

A longitudinal panel setting offers opportunities to investigate the causal relationship between usage and resistance. A fixed-effects approach with usage lagged by 1 or 2 years has shown that increases in usage precede increases in resistance at the national level [19, 20]. These studies, however, cannot explain the long-term impact of antibiotic use on resistance, which is essential to guide policies. Is a rise in resistance owing to usage persistent and widespread or does it fade away?

Reducing antibiotic consumption is often considered a prime solution to slow the dissemination of resistance [21]. If there is a fitness cost to resistance, the absence of selection pressure should lead to a reduction in resistance as susceptible bacteria replace their resistant counterparts [22]. It has also been hypothesized that even if usage is reduced, resistant clones would continue to persist and, if at all, only slowly be outcompeted by their susceptible counterparts [23, 24]. Our research thus analyzes the resistance effects of increases and decreases in antibiotic usage separately over the long term.

We consider the association between usage and resistance as a dynamic relationship and introduce distributed–lag and event study specifications that consistently and efficiently model this dynamic relationship. Our data on antibiotic resistance include all the 26 bacteria-antibiotic (“bug-drug”) combinations tracked by EARS–Net, based on random samples taken from clinical laboratories, and aggregated to a national, annual level, for 26 European countries from 2008 to 2018. We match these data to antibiotic sales data from IQVIA’s MIDAS database, which allows us to estimate the effect of usage on resistance over multiple years. Our hypotheses are that there should be no effect on resistance prior to usage but that resistance in a country will increase with usage.

## Methods

### Antibiotic usage data

Usage data for 26 European countries for the years 2008–2018 is drawn from IQVIA MIDAS database. IQVIA reports the volume of sales of antibiotic molecules used in human medicine based on national surveys. We convert antibiotic sales into Defined Daily Doses (DDDs) using ATC/DDD Index 2020. The World Health Organization defines DDD as the assumed average maintenance dose per day for a drug used for its main indication in adults. We adjust for the population using population estimates from the World Bank DataBank to obtain DDDs per 1000 inhabitants (DpTI).

Antibiotic molecules are active chemical compounds that can be broadly categorized into classes of antibiotics based on their mechanisms of action with bacteria. Using the ATC/DDD index we aggregate DpTI for antibiotics into 12 antibiotic classes, some of which contain only a single antibiotic (Supplementary Table S1). Sales are thus identified by antibiotic class, year, and country. We categorize the 26 European countries into Northern, Southern, Eastern and Western Europe, as detailed in Supplementary Table S2.

Usage levels vary significantly across classes and countries, as shown in Supplementary Figs. S1–S3. To help make the usage data comparable, we convert DpTI into *z*-scores. A *z*-score is defined as the difference between an observation and the sample mean divided by the standard deviation for a class in each country over the years 2008–2018.

### Antibiotic resistance data

The European Antimicrobial Resistance Surveillance System (EARS–Net) collects data on antimicrobial resistance in 8 different bacterial pathogens to 12 antibiotic classes as reported in the Surveillance Atlas of Infectious Diseases. The bacteria-antibiotic combinations covered are detailed in Supplementary Table S3. According to EARS–Net data documentation, all main geographical regions are covered, and, on average, data are considered as representative of the national epidemiology. However, the percentage of population coverage varies among reporting countries and the population under surveillance changes over time.

The data record resistance of specific bacteria—in percentage—by specific antibiotic class, year, and country. We categorize the 8 different bacterial pathogens into Gram-positives and Gram-negatives, as detailed in Supplementary Table S4. Our resistance data therefore vary by country, bacteria-antibiotic class combination, and year from 2008 to 2018, with a range between 0 and 100%, as shown in Supplementary Figs. S4–S7.

### Sample definition

We combine the two datasets using year, country, and antibiotic class to obtain resistance and usage data, for 26 European countries, 11 years, and 26 bacteria-class combinations. We construct a panel on the universe of all units, where a unit is a combination given by country, year, and bacteria-class. Since some countries did not report resistance for every bacteria-class in all years, our panel is an unbalanced one with 6586 observations.

Our main dependent variable is the prevalence of resistance, as given by the percentage of resistant isolates identified by EARS–Net. We use usage *z*-scores or change in *z*-scores, depending on the specification employed, as our main explanatory variable. We select a 6-year panel of the resistance data covering the years 2012–2017 for our analysis. This choice helps set up the event study design with an event horizon running from 1 year prior to 4 years after usage, so that we require usage data from 2008–2018.

### Empirical models

We use three related empirical models to estimate the causal effect of usage on resistance: (1) a Distributed–Lag (D–L) model with fixed effects, (2) a D–L model with first differences and (3) an Event–Study (E–S) model with binned endpoints. Binning refers to the practice of treating the last lag (lead) as an open interval capturing all known changes that have happened (will happen) in the past (future). Our outcome variable is the resistance for a bacteria-class *i*, in country *c*, in year *t*, $R_{i,{{{{{{{\mathrm{c}}}}}}}},t}$. The explanatory variable of interest in model (1) is the *z*-score of usage, happening *j* periods away for a bacteria-class *i*, in country *c*, in year *t*, $U_{i,{{{{{{{\mathrm{c}}}}}}}},t - j}$. The explanatory variable of interest in models (2) and (3) is the change in *z*-score of usage, happening *j* periods away for a bacteria-class *i*, in country *c*, in year *t*, $\Delta U_{i,{{{{{{{\mathrm{c}}}}}}}},t - j}$.

Models (1) and (2) are specified as:

With fixed effects:

1 $$R_{i,{{{{{{{\mathrm{c}}}}}}}},t} = \mathop {\sum}\nolimits_{j = - 1}^4 {\gamma _jU_{i,{{{{{{{\mathrm{c}}}}}}}},t - j} + \mu _i + \mu _c + \theta _t + \varepsilon _{i,{{{{{{{\mathrm{c}}}}}}}},t}}$$

With first differences:

2 $$\Delta R_{i,{{{{{{{\mathrm{c}}}}}}}},t} = \mathop {\sum}\nolimits_{j = - 1}^4 {\lambda _j{{\Delta }}U_{i,{{{{{{{\mathrm{c}}}}}}}},t - j} + \theta _t + \varepsilon _{i,{{{{{{{\mathrm{c}}}}}}}},t}}$$

The event window spans the period from 1 year before usage (or to a change in usage) to 4 years after. We include bacteria-class (*µ*_*i*_) and country (*µ*_*c*_) fixed effects in Model (1), which accounts for unobserved bacteria, class and country effects which are constant across time. In Model (2), first–differencing resistance and usage controls for these effects. For both models, we include year fixed effects (*θ*_*t*_). The error term is given by ***ε***_*i,c,t*_. The coefficients *γ*_*j*_ and *λ*_*j*_ denote the marginal effects of usage on resistance, measuring the slope of these effects from one year to the next. The estimated values of *γ*_*j*_ and *λ*_*j*_ in Models (1) and (2) are expected to differ significantly only if the effect of usage on resistance continues to unfold beyond the 4-year window [25].

Model (3) is specified as:

3 $$R_{i,{{{{{{{\mathrm{c}}}}}}}},t} = \mathop {\sum}\nolimits_{j = - 2}^4 {\beta _jv^{\,j}_{i,{{{{{{{\mathrm{c}}}}}}}},t - j} + \mu _i + \mu _c + \theta _t + \varepsilon _{i,{{{{{{{\mathrm{c}}}}}}}},t}}$$

where the binned variables $v_{{{{{{{{\mathrm{i}}}}}}}},c,t}^{\,j}$ are defined as:

4 $$v_{{{{{{{{\mathrm{i}}}}}}}},c,t}^{\,j} = \left\{ {\begin{array}{*{20}{l}} {\mathop {\sum}\nolimits_{k = - \infty }^{ - 2} {\Delta U_{i,c,t - k} \quad{{{{{{{\mathrm{if}}}}}}}}\;j = - 2} } \\ {\Delta U_{i,c,t - j}\quad\quad\quad\quad{{{{{{{\mathrm{if}}}}}}}} - 2 \, < \, j \, < \, 4} \\ {\mathop {\sum}\nolimits_{k = 4}^\infty {\Delta U_{i,{{{{{{{\mathrm{c}}}}}}}},t - k}\quad\quad\quad{{{{{{{\mathrm{if}}}}}}}}\;j = 4} } \end{array}} \right.$$

Model (3) is a regression of levels on binned changes. The coefficients *β*_*j*_ are the treatment effects, *j* time periods before or after usage, dynamically unfolding over time and are expressed relative to a reference period. The coefficient for the reference period is normalized to zero. Binning the upper and lower endpoints is equivalent to assuming that $\gamma _j = 0$ for all *j* > 4 and for all $j \le - 2$. In other words, this model assumes effects of usage on resistance stay constant for all *j* > 4 and for all $j \le - 2$. Due to the nature and construction of binned variables, the E-S model requires data 2 years prior to the event. These assumptions on the effect window acknowledge that dynamic effects cannot be estimated using the infinite past and the infinite future due to limited data availability and sample restrictions [25].

Model 3 provides readily interpretable coefficients *β*_*j*_ whereas the coefficients *γ*_*j*_ and *λ*_*j*_ from Models 1 and 2 must be linearly transformed to derive the dynamic effects. Statistical properties such as consistency and asymptotic normality are preserved during this linear transformation of D–L model estimates, and variances and covariances of the estimated parameters *γ*_*j*_ and *λ*_*j*_ can be used to recover standard errors for the estimated *β*_*j*_ using a standard linear combination formula [25]. These estimated dynamic treatment effects are unbiased under linear and additive assumptions. Event study models with binned endpoints and D–L models are expected to yield similar treatment effects [25].

The reported dynamic treatment effects estimated using all three models are interpreted as the impact on resistance due to a 1 standard deviation increase in usage. We can observe this impact in the years prior to and after usage. Identification of dynamic treatment effects requires that there be no statistically significant impacts on resistance before any usage happens. Moreover, identification is achieved within bacteria-class and country over time.

We also estimate all the models using segmented data. Specifically, we segment the data by Gram-positive and Gram-negative data to assess whether they respond differently. We also segment by countries, dividing the countries into four regions, West, North, East, and South, to assess whether the same effects are observed in all geographies.

We further segment the data according to whether changes in usage are positive or negative. Using our D–L specifications, with a slight modification, we test for effects on resistance due to positive and negative changes in usage, separately. Our hypothesis is that a positive change in usage will increase resistance whereas a negative change in usage will decrease resistance. Our empirical model to assess this hypothesis is given by

5 $$R_{i,{{{{{{{\mathrm{c}}}}}}}},t} = \mathop {\sum}\nolimits_{j = - 1}^4 {\gamma _jD_{i,{{{{{{{\mathrm{c}}}}}}}},t - j} + \mu _i + \mu _c + \theta _t + \varepsilon _{i,{{{{{{{\mathrm{c}}}}}}}},t}}$$

where $D_{i,{{{{{{{\mathrm{c}}}}}}}},t - j}$ is an indicator variable that indicates whether there was a positive or negative change above a specific threshold in the usage of antibiotics treating bacteria-class *i*, in country *c*, *j* periods away.

## Results

**Fig. 1:**
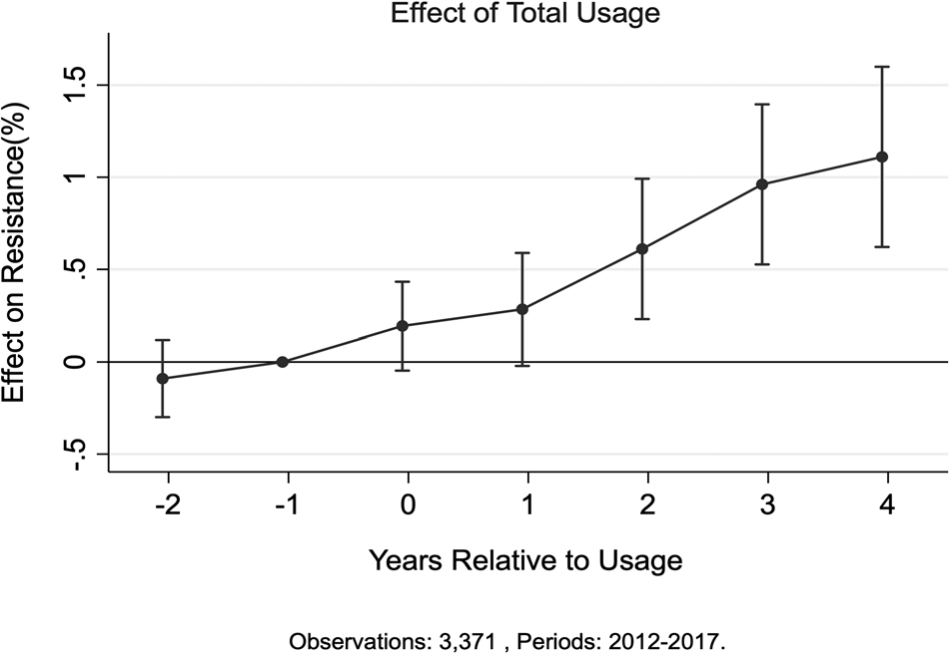
General characterization of resistance effects. The figure plots coefficients from fixed-effect estimation methodology along with the 95% confidence intervals, for a 1 s.d. increase in usage. See Supplementary Fig. S8 for a plot using coefficients from all three estimation methodologies. The effect on resistance is normalized to zero in the year prior to usage. Two years prior to usage, the resistance effect is not statistically different from zero, suggesting no pre-trend. The coefficients of interest for different empirical specifications are reported in Table 1

It should be noted, for ease of viewing, only estimates from Model (1) are presented in the following sections. Corresponding results from all three models are presented in the supplementary material as Supplementary Fig. S8. We start our analysis with a general characterization of resistance effects. The results are shown in Fig. 1, which shows the average effect of a 1 standard deviation (1 s.d.) increase in usage of a specific antibiotic class on resistance in a targeted bacterial species. On average, a 1 s.d. increase in a year corresponds to an increase of 140 defined daily doses per thousand inhabitants (Table 1). Using our entire sample, we find that in year “0” (the year of usage), there is an immediate but statistically non-significant positive effect on resistance compared to the prior year. The effect on resistance unfolds gradually, with a positive and increasing trend for at least 4 years after usage. A 1 s.d. increase in usage increases resistance by about 0.6% (1%) 2 (4) years after use. In addition, as expected, we find no effect on resistance that pre-dates usage (i.e., in period *t*–2). We carry out numerous sensitivity analysis, including longer effect window, other control variables, and placebo tests, as discussed in the Robustness Checks section below. A 1% increase in resistance from, say, the average level of 24–25% is not clinically significant, but is problematic if it is part of an ongoing process in which resistance builds year after year.

**Table 1:** Average, standard deviation, antibiotic usage and resistance per year are presented

	Usage (DDD per thousand inhabitants)		Resistance	
Year	Average (by drug and country)	Average s.d. (by drug and country)	Average	Std. Dev.
2008	494.48	152.58	**20.92**	**22.57**
2009	534.57	154.28	**21.97**	**22.96**
2010	354.15	154.17	**22.00**	**23.74**
2011	343.58	148.71	**22.41**	**23.92**
2012	305.17	133.79	24.56	25.29
2013	283.89	133.19	24.71	25.4
2014	276.33	132.39	25.09	26.02
2015	291.26	132.99	24.85	25.65
2016	287.14	132.79	25.19	26.05
2017	287.69	133.39	24.79	25.84
2018	256.40	137.10	**24.55**	**25.48**

The third column presents what a 1 standard deviation increase in usage per year corresponds to in DDD per thousand inhabitants

The analysis does not use data from the bold values, which are provided only for context. The decreases in usage (average and standard deviation) observed around 2012 are due to the addition of antibiotics with low volume into the data

**Fig. 2:**
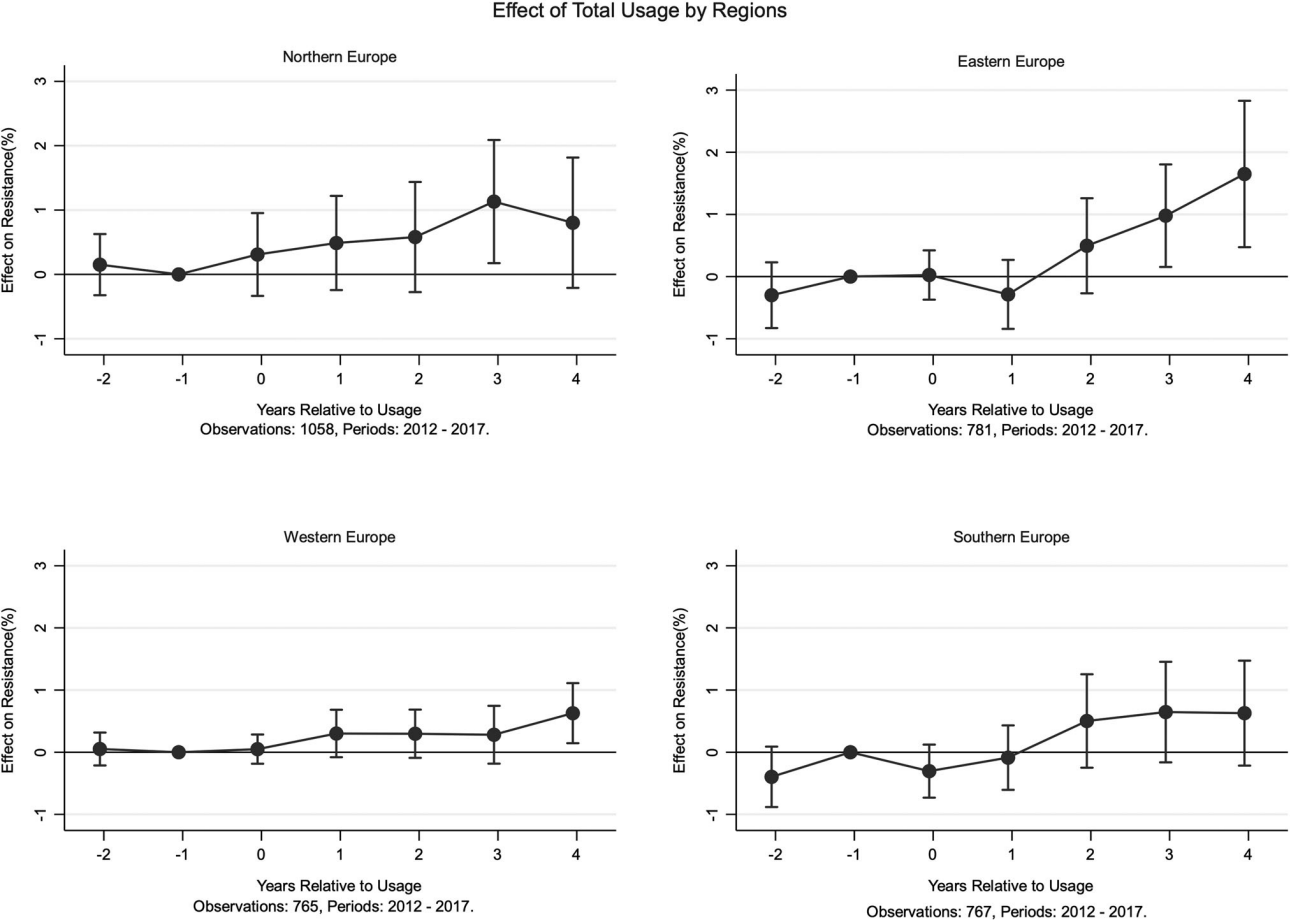
Resistance in four European regions. This figure plots the coefficients from fixed-effect estimation methodology along with the 95% confidence intervals. See Supplementary Fig. S10 for a plot using coefficients from all three estimation methodologies. The effect on resistance is normalized to zero in the year prior to usage

The nature of our data allows us to go beyond the general characterization of resistance. We categorize the 26 European countries into Northern, Southern, Eastern and Western Europe and carry out our analysis by region. Figure 2 presents results for the four regions. We find a positive and persistently increasing trend in all four regions. The magnitude and significance of the effect varies across regions. Eastern Europe appears to be most sensitive, with a 1 s.d. increase in usage being followed by an increase of 1.6% in the prevalence of resistance after 4 years.

**Fig. 3:**
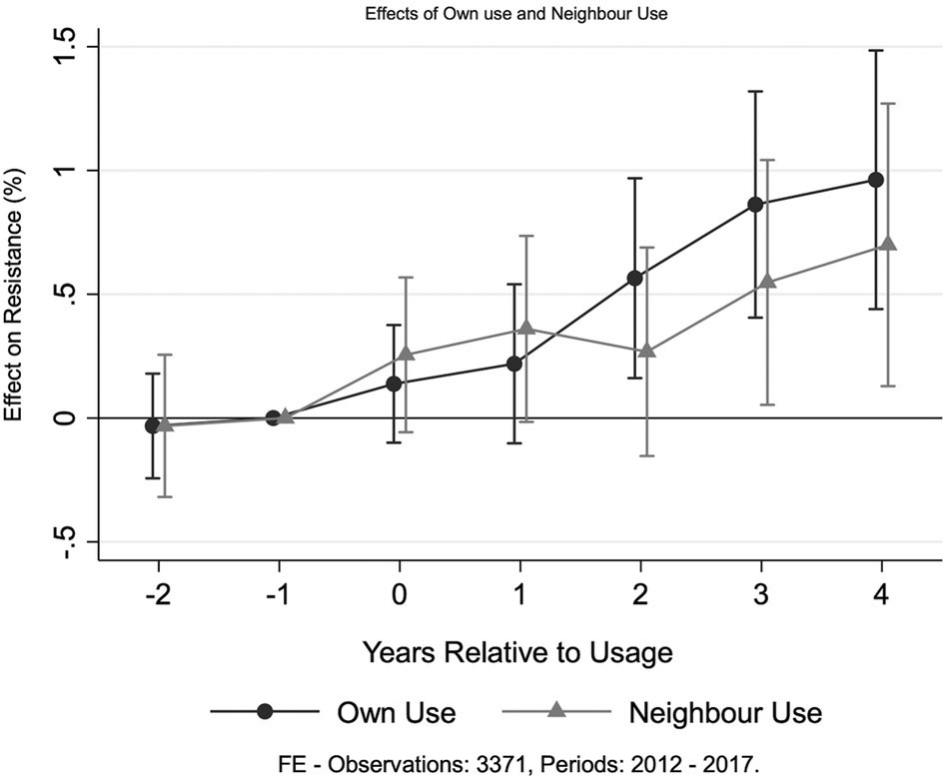
Resistance effects for own and neighbor use. The figure plots the coefficients from fixed-effect estimation methodology along with the 95% confidence intervals. The effect on resistance is normalized to zero in the year prior to usage. Resistance effects arising from own usage are similar to Fig. 1. In addition, we find evidence of neighbors’ use driving resistance effects

In addition, we explore resistance effects arising from usage in surrounding countries. To do this, we calculated, for each country and drug, the sum of use of that drug in all other countries in the region (“neighbors”). We then include distributed lags and a lead of the *z*-score of total neighbors’ usage in our empirical specifications along with all other variables mentioned earlier. We use the four European regions described above. Figure 3 illustrates the effect of own and neighbors’ usage on the prevalence of resistance. The estimated effect on resistance from own use remains almost unchanged from Fig. 1. Of particular interest, however, we find evidence of neighbors’ usage driving resistance.

**Fig. 4:**
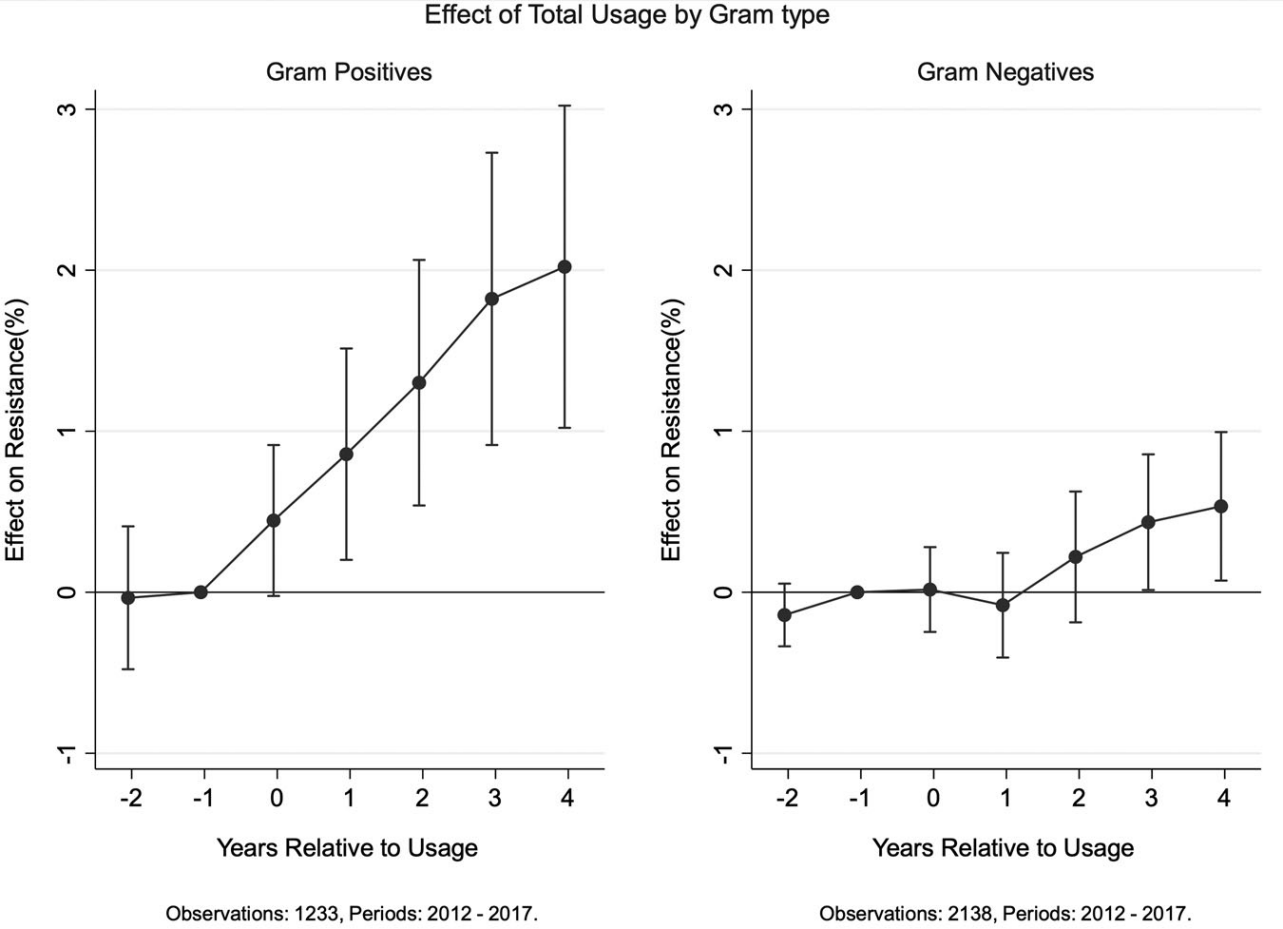
Resistance effects across Gram types. The figure plots the coefficients from fixed-effect estimation methodology along with the 95% confidence intervals. See Supplementary Fig. S11 for plots using coefficients from all three estimation methodologies. The effect on resistance is normalized to zero in the year prior to usage. Resistance appears to be more sensitive to usage among Gram-positive bacteria

We also compare across bacteria, which can be classified into Gram-negative and Gram-positive. We plot the dynamic effects on resistance across both groups in Fig. 4. We find the effect on resistance is strongest among Gram-positive bacteria—a 1 s.d. increase in usage (i.e., a *z*-score of 1) increases resistance significantly starting in the year of use. Resistance continues to rise and by 4 years it increases by approximately 1.5–2% points. For Gram-negative bacteria, we find the dynamic effects are much smaller, with significant impacts starting only 3 years after use.

We use change in *z*-scores to represent the change in usage and segment the positive and negative changes into two samples [26, 27]. Increases and decreases are defined by specific thresholds and are analyzed in separate regressions. The mean positive change in usage *z*-scores is 0.60 and the mean negative change is –0.58. We use these averages to set our threshold levels.

**Fig. 5:**
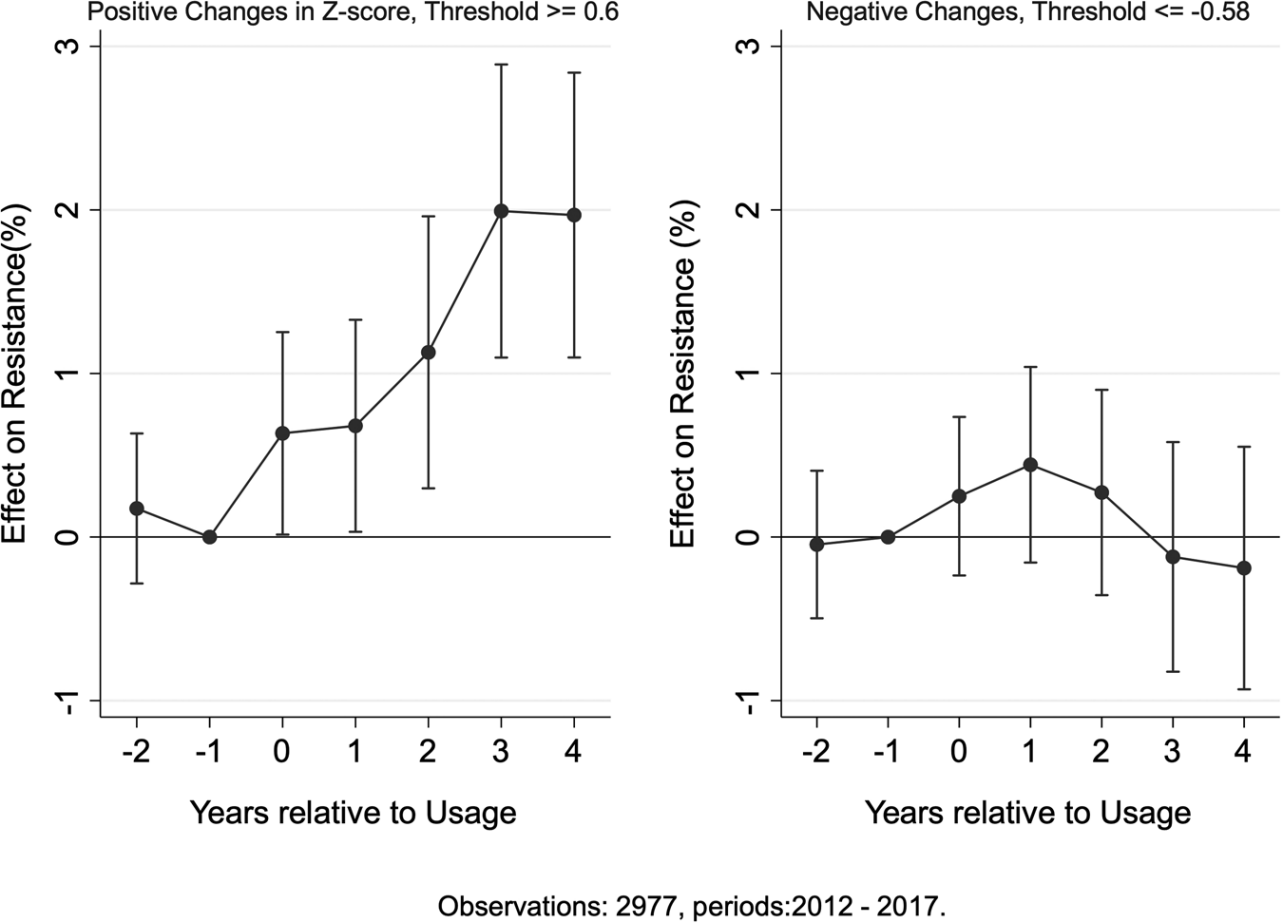
Resistance effects for increase vs. decrease in usage. These figures plot the coefficients and 95% confidence intervals from two separate regressions for increase and decrease in usage. A change in usage *z*-score $\ge 0.60$ ($\le - 0.58$) is defined as an increase (decrease) in use. Fixed-effects estimation methodology is used here. We observe a significant response of resistance only to increases in usage

Increases in usage above the positive threshold immediately and persistently increase resistance. In contrast, decreases in usage have little to no observable effect on resistance. In other words, reversibility of resistance due to a decrease in usage is at best slow. These results are illustrated in Fig. 5.

### Robustness checks (sensitivity analysis)

The length of the effect window chosen helps identify dynamic treatment effects and secular time-fixed effects separately. In other words, the chosen effect window directly affects identification. To illustrate the positive trend is not due to our choice of the effect window we plot Supplementary Fig. S9, which plots the event study estimates from Eq. (1), using usage, for 3 years prior to use and 1, 2, 3, 4, and 5 years after use. Note that increasing the effect window decreases the number of years for which we observe resistance which in turn reduces the number of observations used for estimation. The trend is always similar regardless of the number of years. For all our results, we chose to report estimates 1 year prior to and 4 years after usage. This allows us to identify the resistance effects for at least 6 years (2012–2017) using usage for 11 years (2008–2018).

Another concern is the possibility that usage responds to national factors which in turn affect resistance. To test for violations in our identifying assumptions we use national factors as controls in our event study specifications. We follow Collignon et al., Klein et al., and Adda, in choosing variables representing national factors and carry out the test using development indicators such as annual GDP per capita growth, corruption perception index, health expenditure per capita, access to basic sanitation, and percentage of urban population [9, 19, 28]. Adding these variables as controls in all three empirical specifications—Eqs. (1), (2) and (3)—does not materially change the estimated coefficients on usage or their significance, as shown in Table 2 and Supplementary Fig. S12.

**Table 2:** The table reports the *β*_*j*_ coefficients for fixed effects (FE), first differences (F-D) and event-study with binned endpoints (E-S)

	FE	FE with controls	F-D	E-S
*t* + 1	–0.0901 (0.107)	–0.0313 (0.118)	–0.0123 (0.102)	–0.0617 (0.119)
*t*	0.193 (0.122)	0.152 (0.127)	0.233 (0.128)	0.204 (0.134)
*t* – 1	0.284 (0.156)	0.325 (0.170)	0.357 (0.190)	0.364* (0.170)
*t* – 2	0.611** (0.194)	0.630** (0.210)	0.673** (0.241)	0.630** (0.209)
*t* – 3	0.961*** (0.222)	0.999*** (0.243)	0.922** (0.282)	0.996*** (0.236)
*t* – 4	1.110*** (0.249)	1.128*** (0.271)	1.033*** (0.313)	1.170*** (0.264)
Observations	3371	3016	2711	2873

Coefficients for fixed effects with controls are also reported

Standard errors in parentheses; standard errors clustered at country and bacteria-class level; *p < 0.05, **p < 0.01, ***p < 0.001

Furthermore, we conduct placebo tests using the same national factors as left-hand side variables in our event study specifications. Usage should not have any impact on these national factors. Therefore, we should expect to find noisy insignificant estimates both before and after usage. Supplementary Fig. S13 illustrates the effects on national factors; none is persistently significantly different from zero. These placebo tests help us probe the robustness of our research design by checking for associations between variables that should not be present if the research design is sound.

## Discussion

This paper studies the long-term effect of mass use of antibiotics on antibiotic resistance. Alongside the average treatment effect on resistance across 26 countries and 26 bug-drug combinations, we also isolate the effect for four European regions and two Gram-types. We observe an immediate increase and a persistent upward trend in resistance following usage that continues for at least 4 years after antibiotic usage. The consistency of results from the three different empirical models suggests robustness. In addition, we demonstrate an empirical methodology to analyze increases and decreases in usage separately. This reveals that the effect on resistance from a decrease in usage is insubstantial relative to the effect from an increase in usage. Our findings stress the need for judicious usage.

One challenging finding is that the prevalence of resistance in a country rises following increased usage in neighboring countries, controlling for usage in that country. This emphasizes the international dimension of the antibiotic resistance problem and suggests a role for international cooperation.

Our study has numerous limitations. There are several factors at play that drive the emergence of resistance against antibiotics among bacteria. The molecular mechanisms behind the emergence of resistant bacteria are ancient and the natural concentration of antibiotics and resistant genes in the environment could lead to novel resistance mechanisms against antibiotic compounds [22]. Intensive livestock rearing, aquaculture, and agriculture rely heavily on usage of antibiotics, promoting the emergence and spread of resistant bacteria. Ideally, one would consider all these factors in determining how resistance evolves over time, though appropriate indicators to measure the role of environmental factors are limited and there is a disconnect in surveillance data for humans and animals [29, 30]. Furthermore, given the nature of our data on use and resistance, we were unable to trace the mechanisms through which resistance spreads. Importantly, the empirical design of our study does not allow us to identify the effect of persistent but constant usage of antibiotics; instead, it informs us that resistance is sensitive to variation in usage and that increases in resistance have a persistent impact on resistance.

The variation in the sensitivity of resistance to use across classes of bacteria (Gram-positive and -negative) and across regions suggests that we should be cautious about the external validity of this analysis. However, it is revealing that we observe an increase in resistance in all four regions of Europe following an increase in usage, so the general result appears to be robust at least within the range of resistance levels seen in Europe. Moreover, the methodology applied here can be used in studies in other regions, and across other time-varying risk factors.

Numerous previous studies have used cross-sectional data that do not allow for the exclusion of potential endogeneity [5–9]. In addition, most studies have attempted to find a single estimate to describe the relationship. A single estimate is simple and describes the relationship across 1 or 2 years, but it cannot reveal the evolution of resistance [19, 20]. Moreover, studies with high-frequency data focused on short-run seasonal effects and were not designed to find long-run associations [10, 11]. We have added to this literature a consistent methodology to model and estimate the dynamic relationship between usage and the prevalence of resistance over time. Our model allows us to control for confounding factors and using a panel setting we also control for unobserved time-invariant heterogenous effects. Existing results suggest a static positive relationship between usage and resistance. We estimate a dynamic positive relationship, and the key empirical finding is that increased antibiotic usage has persistent effects on the prevalence of resistance lasting for several years at least. The consistency of results from the three different empirical models suggests robustness. Furthermore, using national surveillance data on 26 bug-drug combinations, we find that resistance is more sensitive to increases than to decreases in usage, consistent with the findings of Aliabadi et al. [12].

Since usage is continuous and has become a core part of modern medical practices, we infer from our results that the resistance crisis will likely worsen over time. In these circumstances, prudent use and stewardship of antibiotics is of particular importance. The recent documented increase in the prescription of antibiotics owing to the COVID-19 pandemic is likely to lead to a long-term increase in resistance [31, 32]. Our findings suggest that there is a strong case for policies to discourage overuse of antibiotics, but also that there is likely to be a need to support the development of new antibiotics.

Decreasing usage to mitigate resistance is a commonly proposed solution and advocates draw the attention of policymakers to reduce antibiotic usage significantly [28]. However, in our results, decrease in usage only slowly decreases resistance and there was no evidence of a reversal of resistance. Therefore, reducing usage is not a complete solution to alleviating high levels of resistance, at least in the short run.

## Supplementary information

Supplementary Material
