# Supplementary material for: Persistence of resistance: a panel data analysis of the effect of antibiotic usage on the prevalence of resistance
Source: J Antibiot (Tokyo). 2023 Feb 28;76(5):270–8. doi: 10.1038/s41429-023-00601-6 (PMC9970858; doi:10.1038/s41429-023-00601-6)
Supplement: Supplementary file 1 — Supplementary Material [file 41429_2023_601_MOESM1_ESM.docx]

**Supplemental Material for**

Persistence of Resistance: A panel data analysis of the effect of antibiotic usage on the prevalence of resistance

Sakib Rahman^1^, Aaron S. Kesselheim^2^, Aidan Hollis^1^*

^1^ Department of Economics, University of Calgary; Calgary, AB, Canada.

^2^ Program on Regulation, Therapeutics, And Law, Division of Pharmacoepidemiology and Pharmacoeconomics, Department of Medicine, Brigham and Women’s Hospital and Harvard Medical School; Boston, MA, USA.

* Corresponding author, Aidan Hollis.

Email: ahollis@ucalgary.ca ; Telephone: +1 (403) 220-5861

**This document includes:**

Descriptive Statistics on the data

Figures S1 to S13

Tables S1 to S6

Descriptive Statistics of the Data

**Antibiotic Usage Data**

Figure S1 presents average usage in DpTIs (Daily Defined Doses per Thousand Inhabitants) for 2015 across these countries. Figure S2 plots the average usage in DpTI across antibiotic classes. Panel A includes four of the most used antibiotics, where Aminopenicillins lead in both retail and hospital use. Panel B shows the usage for the rest of the classes. The volume of use for antibiotic classes in Panel B is much lower than the antibiotic classes in Panel A. Figure S3 plots the average usage in DpTI for the four European regions over time. Southern Europe has much higher usage than the other regions.

These plots, along with summary statistics (Table S5), clearly demonstrate the significant variations in usage per class amongst countries and sectors, which motivate the conversion of usage data into z-scores.

From 2008-2018 there are 2,979 positive changes and 3,128 negative changes in usage across 26 European countries. Figure S8 (which excludes outliers) shows the distribution of usage changes using both DpTI and z-scores. The average positive (negative) change in usage is 1·70 DpTI (-1·79 DpTI).

**Antibiotic Resistance Data**

EARS–Net includes only data from invasive blood and cerebrospinal fluid isolates. This restriction minimizes inconsistencies. Resistance is assessed based on the ability of antibiotics, at specified concentrations, to inhibit bacterial growth, using clinical thresholds established by the European Committee on Antimicrobial Susceptibility Testing, and bacteria are categorized as susceptible, susceptible with increased exposure, or resistant to each of the relevant antibiotics. Moreover, percentages of non-susceptible and resistant isolates are not calculated if results from AST for a specific bacteria-antibiotic class were reported for fewer than ten isolates in a country in a year.

The distribution of resistance is shown in Figure S4. Figure S5 plots average resistance over time for each bacterium across countries. On average, Enterococcus faecium exhibits the highest occurrence of resistance followed by Acinetobacter spp. When we plot the average resistance of the relevant bacteria in relationship to the antibiotic classes which are used to treat them (Figure S6), we observe high levels of resistance for two of the heavily used antibiotic class (Aminopenicillins and Fluoroquinolones). For little-used antibiotic classes (except for high-level Gentamicin), resistance tends to be low. Figure S7 reveals an increasing trend in average resistance for Eastern and Southern Europe but a relatively flat trend for Northern and Western Europe. Occurrence of resistance is high for both Eastern and Southern Europe and low for both Northern and Western Europe. Summary statistics for resistance by region and bacteria-class combination are also provided in Table S6.

Data are available for Acinetobacter spp. starting only in 2012, and not all countries report resistance data on this bacterium. Moreover, a few smaller countries (e.g., Croatia, Estonia, Latvia, and Slovakia) have missing resistance data for few bacteria-class combinations. This makes our panel an unbalanced one.


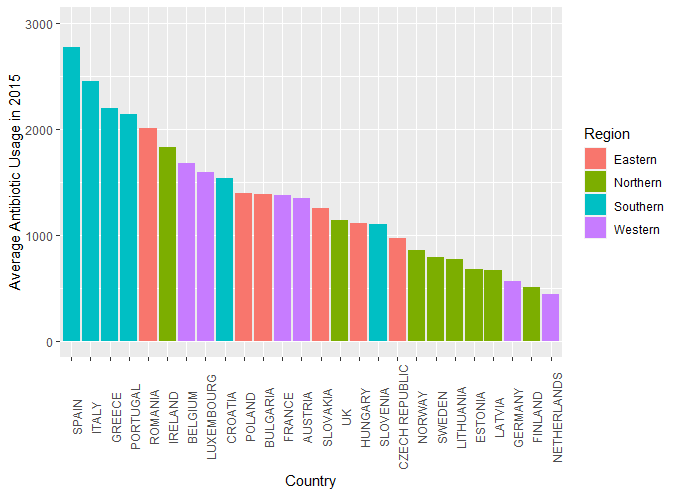


Figure S1. Snapshot of Antibiotic Usage across 26 European countries in 2015.

The average antibiotic usage in 2015, represented by DDD/1000 inhabitants, is on the y-axis. The x-axis shows countries, colored according to their regions.


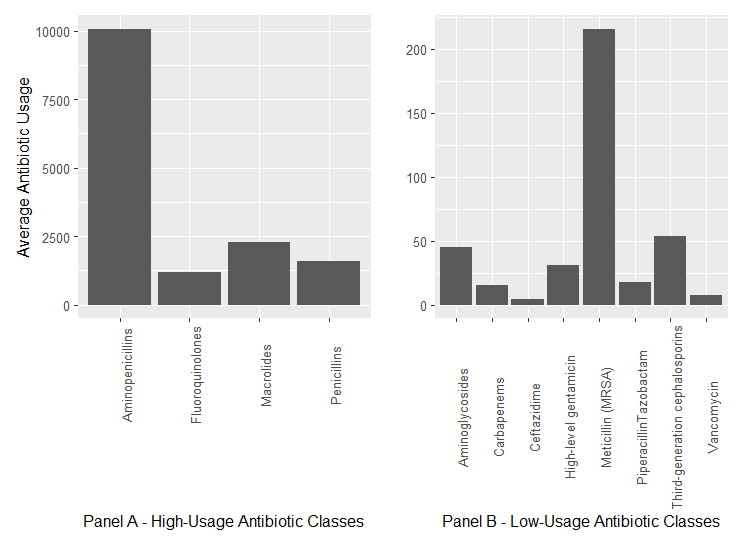


Figure S2. Average Antibiotic Usage by class of antibiotics.
The average antibiotic usage, represented by DDD/1000 inhabitants, is on the y-axis. The x-axis shows antibiotic classes.


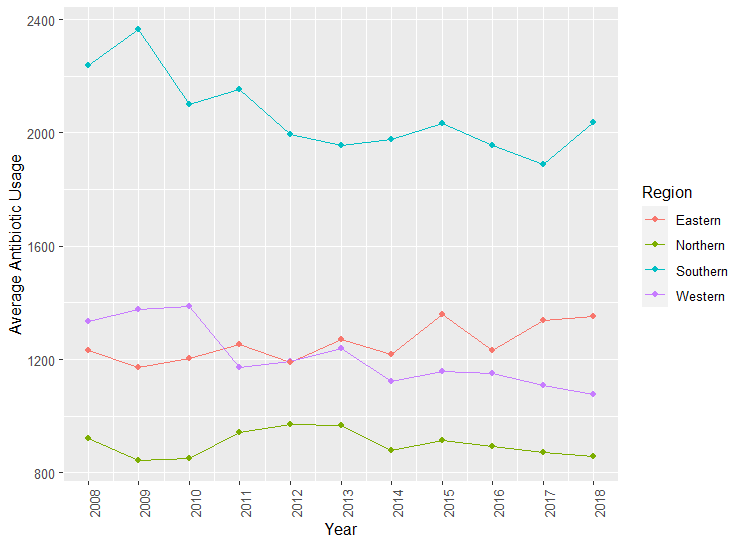


Figure S3. Average Antibiotic Usage across 4 broad European Regions.

The average antibiotic usage, represented by DDD/1000 inhabitants, is on the y-axis. The x-axis shows years.

Figure S4. Distribution of Antibiotic Resistance across bacteria.

The probability for antibiotic resistance to take on a given value is given on the y-axis. The values that resistance level takes in our data is on the x-axis.


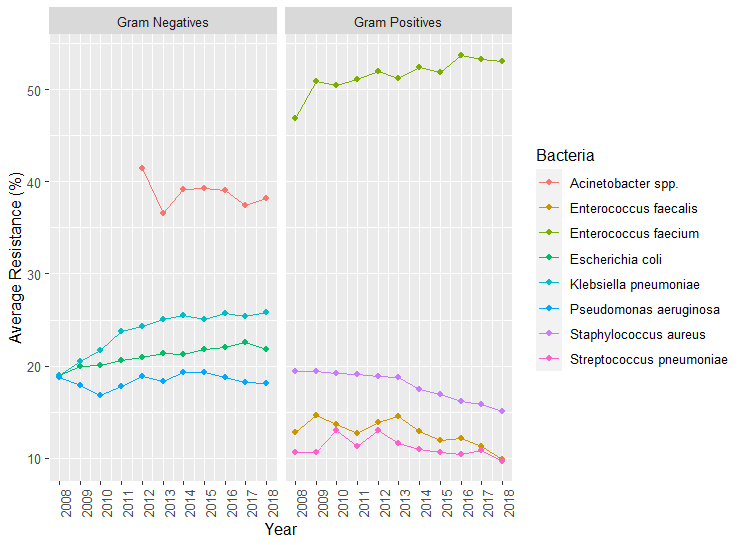


Figure S5. Average Antibiotic Resistance across bacteria.
The average antibiotic resistance in % is on the y-axis. The x-axis shows years. Bacteria are categorized into two panels according to their Gram types.


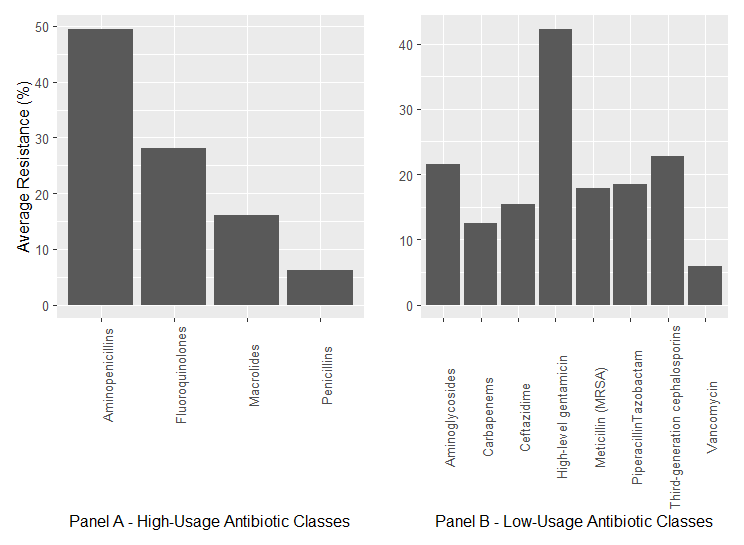


Figure S6. Average Antibiotic Resistance across antibiotic classes.
The average antibiotic resistance in % is on the y-axis. The x-axis shows the antibiotic class.


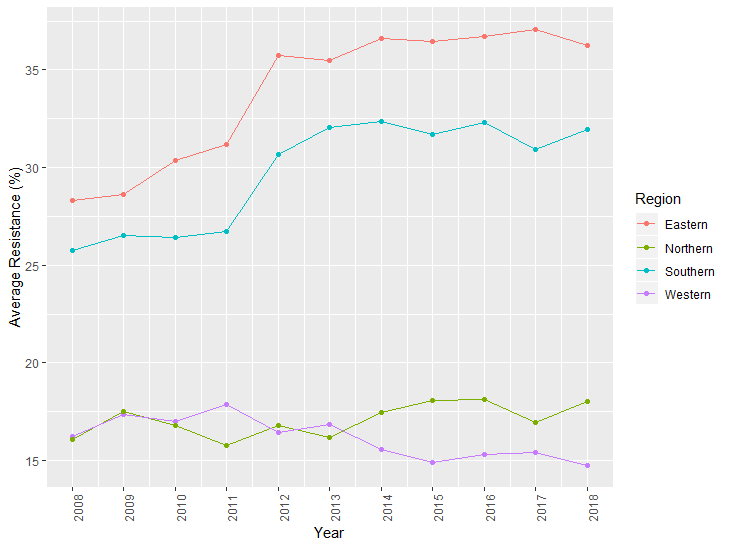
Figure S7. Average Antibiotic Resistance across 4 broad European Regions.
The average antibiotic resistance in % is on the y-axis. The x-axis shows years. For a given region, resistance level is available for specific countries, specific class-bacteria combination and 11 years.

Figure S8. General characterization of antibiotic resistance effects.

This figure plots coefficients from three estimation methodologies along with the 95% confidence intervals. The effect on resistance is normalized to zero in the year prior to usage. Two years prior to usage, the resistance effect is not statistically different from zero suggesting no pre-trend. The coefficients of interest for the fixed effects empirical specification are reported in Table 1.

**Figure S9. Resistance Effects using Different Effect Windows.**

This figure plots coefficients from Distributed-Lag model with Fixed Effects. We use different effect windows, precisely different lag and lead lengths. The trend is increasing and persistent as our main findings.

Figure S10. Resistance across 4 European regions.
This figure plots coefficients from three estimation methodologies along with the 95% confidence intervals. The effect on resistance is normalized to zero in the year prior to usage. Two years prior to usage, the resistance effect is not statistically different from zero suggesting no pre-trend.

Figure S11. Resistance effects across Gram types.
This figure plots coefficients from three estimation methodologies along with the 95% confidence intervals. The effect on resistance is normalized to zero in the year prior to usage. Two years prior to usage, the resistance effect is not statistically different from zero suggesting no pre-trend.

Figure S12. Resistance effects using national level confounders.

We carry out the estimations, including controls, using all three specifications. For control variables, we include measures of GDP, Corruption, Population Density, Health Expenditure per capita, Access to Basic and Safe Sanitation and Number of Physicians per capita. The results are not different from our main results in the paper, presented in Figs. 1 and S8.

Figure S13. Placebo Tests to check the soundness of the empirical model

The model should not predict a relationship between usage and national level confounders, i.e., usage does not influence GDP, CPI and other national level factors and if the model is specified correctly, we should not see any relationship and significance between usage and these factors. All predicted effects are insignificant.

Table S1. List of Antibiotic Molecules

| Antibiotic Molecules and their relevant Antibiotic Classes | |
| --- | --- |
| **Antibiotic Molecule** | **Antibiotic Class** |
| Gentamicin, Netilmicin, Tobramycin | Aminoglycosides |
| Ampicillin, Amoxicillin | Aminopenicillins |
| Meropenem, Imipenem | Carbapenems |
| Ceftazidime | Ceftazidime |
| Ciprofloxacin, Levofloxacin, Moxifloxacin, Norfloxacin, Ofloxacin | Fluoroquinolones |
| Gentamicin | Gentamicin |
| Erythromycin, Clarithromycin, Azithromycin | Macrolides |
| Cefoxitin, Cloxacillin, Dicloxacillin, Flucloxacillin, Oxacillin | Methicillin |
| Oxacillin, Penicillin V, Penicillin G | Penicillin |
| Piperacillin | Piperacillin |
| Cefotaxime, Ceftriaxone, Ceftazidime | Third gen cephalosporins. |
| Vancomycin | Vancomycin |

Note: Some antibiotics constitute their own class for the purpose of this analysis.

Table S2. List of Countries categorized into 4 Regions

| Countries | Regions |
| --- | --- |
| Estonia, Finland, Ireland, Latvia, Lithuania, Norway, Sweden, and UK | Northern Europe |
| Croatia, Greece, Italy, Portugal, Slovenia, and Spain | Southern Europe |
| Bulgaria, Czech Republic, Hungary, Poland, Romania, and Slovakia | Eastern Europe |
| Austria, Belgium, France, Germany, Luxembourg, and Netherlands | Western Europe |

Table S3. List of Bacteria-Antibiotic Combinations

Our resistance data include bacteria-class combinations for the categories marked with an X; resistance data is unavailable for other bacteria-class combinations.

| **Combinations of Bacteria and Antibiotic Classes** | | | | | | | | | |
| --- | --- | --- | --- | --- | --- | --- | --- | --- | --- |
| **Antibiotic Class** | **Bacteria** | | | | | | | | |
|  |  | Acinetobacter spp | Enterococcus faecalis | Enterococcus faecium | Escherichia coli | Klebsiella pneumoniae | Pseudomonas aeruginosa | Staphylococcus aureus | Streptococcus pneumoniae |
|  | Aminoglycosides | **x** |  |  | **x** | **x** | **x** |  |  |
|  | Aminopenicillins |  | **x** | **x** | **x** |  |  |  |  |
|  | Carbapenems | **x** |  |  | **x** | **x** | **x** |  |  |
|  | Ceftazidime |  |  |  |  |  | **x** |  |  |
|  | Fluoroquinolones | **x** |  |  | **x** | **x** | **x** |  |  |
|  | Gentamicin |  | **x** | **x** |  |  |  |  |  |
|  | Macrolides |  |  |  |  |  |  |  | **x** |
|  | Methicillin |  |  |  |  |  |  | **x** |  |
|  | Penicillin |  |  |  |  |  |  |  | **x** |
|  | Piperacillin |  |  |  |  |  | **x** |  |  |
|  | 3d-gen ceph. |  |  |  | **x** | **x** |  |  |  |
|  | Vancomycin |  | **x** | **x** |  |  |  |  |  |

Table S4. List of Bacterial Pathogens categorized into Gram Positive and Negative

| **Bacterial Pathogens** | **Gram Type** |
| --- | --- |
| Acinetobacter spp., Escherichia Coli, Klebsiella Pneumoniae and Pseudomonas Aeruginosa | Gram-Negative |
| Enterococcus Faecalis, Enterococcus Faecium, Staphylococcus Aureus and Streptococcus Pneumoniae. | Gram-Positive |

Table S5. Summary Statistics for Antibiotic Usage by region and class.

| Class | Region | Total Use | Average Use | Std.Dev. |
| --- | --- | --- | --- | --- |
| Aminoglycosides | Eastern | 4,388 | 69 | 73 |
| Aminopenicillins | Eastern | 589,073 | 9,204 | 3,621 |
| Carbapenems | Eastern | 682 | 11 | 6 |
| Ceftazidime | Eastern | 337 | 5 | 6 |
| Fluoroquinolones | Eastern | 88,156 | 1,377 | 520 |
| High-level gentamicin | Eastern | 3,129 | 49 | 48 |
| Macrolides | Eastern | 153,094 | 2,392 | 585 |
| Meticillin (MRSA) | Eastern | 3,903 | 93 | 154 |
| Penicillins | Eastern | 89,823 | 1,403 | 1,268 |
| PiperacillinTazobactam | Eastern | 169 | 3 | 1 |
| 3^rd^ Gen cephalosporins | Eastern | 5,880 | 92 | 82 |
| Vancomycin | Eastern | 266 | 4 | 2 |
| Aminoglycosides | Northern | 2,616 | 30 | 28 |
| Aminopenicillins | Northern | 459,490 | 5,221 | 3,863 |
| Carbapenems | Northern | 1,015 | 13 | 6 |
| Ceftazidime | Northern | 280 | 3 | 3 |
| Fluoroquinolones | Northern | 55,541 | 631 | 119 |
| High-level gentamicin | Northern | 1,900 | 22 | 29 |
| Macrolides | Northern | 157,605 | 1,791 | 1,172 |
| Meticillin (MRSA) | Northern | 36,867 | 455 | 483 |
| Penicillins | Northern | 207,602 | 2,359 | 2,727 |
| PiperacillinTazobactam | Northern | 1,737 | 22 | 17 |
| 3^rd^ Gen cephalosporins | Northern | 2,539 | 29 | 19 |
| Vancomycin | Northern | 621 | 7 | 6 |
| Aminoglycosides | Southern | 3,928 | 61 | 152 |
| Aminopenicillins | Southern | 1,145,868 | 17,904 | 6,233 |
| Carbapenems | Southern | 1,277 | 20 | 12 |
| Ceftazidime | Southern | 299 | 5 | 4 |
| Fluoroquinolones | Southern | 112,014 | 1,750 | 718 |
| High-level gentamicin | Southern | 2,931 | 46 | 151 |
| Macrolides | Southern | 213,765 | 3,340 | 2,493 |
| Meticillin (MRSA) | Southern | 6,203 | 98 | 131 |
| Penicillins | Southern | 76,006 | 1,188 | 1,876 |
| PiperacillinTazobactam | Southern | 1,315 | 21 | 14 |
| 3^rd^ Gen cephalosporins | Southern | 3,526 | 55 | 46 |
| Vancomycin | Southern | 550 | 9 | 5 |
| Aminoglycosides | Western | 1,657 | 26 | 32 |
| Aminopenicillins | Western | 610,911 | 9,697 | 4,664 |
| Carbapenems | Western | 1,200 | 20 | 13 |
| Ceftazidime | Western | 390 | 6 | 4 |
| Fluoroquinolones | Western | 78,366 | 1,244 | 370 |
| High-level gentamicin | Western | 694 | 11 | 18 |
| Macrolides | Western | 114,312 | 1,814 | 815 |
| Meticillin (MRSA) | Western | 6,586 | 105 | 81 |
| Penicillins | Western | 67,317 | 1,069 | 1,520 |
| PiperacillinTazobactam | Western | 1,495 | 27 | 17 |
| 3^rd^ Gen cephalosporins | Western | 2,929 | 46 | 31 |
| Vancomycin | Western | 552 | 11 | 4 |

Notes: Total Use indicates the total usage in all countries in a region over all years. Average use indicates the average use in year in a country and Std.Dev. indicates the standard deviation of usage calculated by country.

Table S6a. Summary Statistics for Resistance by Antibiotic Class and Bacteria in Eastern Europe.

AvgRest, SDRest, MinRest, MaxRest stands for Average Resistance, Standard Deviation of Resistance, Minimum Resistance and Maximum Resistance, respectively.

| Region | Class | Bacteria | Gram | AvgRest | SDRest | MinRest | MaxRest |
| --- | --- | --- | --- | --- | --- | --- | --- |
| Eastern | Aminopenicillins | Enterococcus faecalis | Positive | 3 | 4 | 0 | 21 |
| Eastern | High-level gentamicin | Enterococcus faecalis | Positive | 44 | 8 | 26 | 76 |
| Eastern | Vancomycin | Enterococcus faecalis | Positive | 1 | 1 | 0 | 4 |
| Eastern | Aminopenicillins | Enterococcus faecium | Positive | 95 | 6 | 63 | 100 |
| Eastern | High-level gentamicin | Enterococcus faecium | Positive | 68 | 10 | 45 | 86 |
| Eastern | Vancomycin | Enterococcus faecium | Positive | 14 | 12 | 0 | 40 |
| Eastern | Aminoglycosides | Escherichia coli | Negative | 16 | 7 | 6 | 36 |
| Eastern | Aminopenicillins | Escherichia coli | Negative | 64 | 6 | 53 | 83 |
| Eastern | Carbapenems | Escherichia coli | Negative | 0 | 1 | 0 | 3 |
| Eastern | Fluoroquinolones | Escherichia coli | Negative | 31 | 7 | 18 | 44 |
| Eastern | Third-generation cephalosporins | Escherichia coli | Negative | 21 | 10 | 2 | 42 |
| Eastern | Aminoglycosides | Klebsiella pneumoniae | Negative | 52 | 11 | 26 | 71 |
| Eastern | Carbapenems | Klebsiella pneumoniae | Negative | 5 | 8 | 0 | 32 |
| Eastern | Fluoroquinolones | Klebsiella pneumoniae | Negative | 51 | 13 | 11 | 71 |
| Eastern | Third-generation cephalosporins | Klebsiella pneumoniae | Negative | 58 | 13 | 35 | 81 |
| Eastern | Aminoglycosides | Pseudomonas aeruginosa | Negative | 33 | 12 | 14 | 63 |
| Eastern | Carbapenems | Pseudomonas aeruginosa | Negative | 33 | 15 | 9 | 70 |
| Eastern | Ceftazidime | Pseudomonas aeruginosa | Negative | 29 | 12 | 11 | 66 |
| Eastern | Fluoroquinolones | Pseudomonas aeruginosa | Negative | 36 | 11 | 18 | 62 |
| Eastern | PiperacillinTazobactam | Pseudomonas aeruginosa | Negative | 32 | 11 | 11 | 62 |
| Eastern | Macrolides | Streptococcus pneumoniae | Positive | 21 | 11 | 3 | 48 |
| Eastern | Penicillins | Streptococcus pneumoniae | Positive | 12 | 13 | 0 | 61 |
| Eastern | Meticillin | Staphylococcus aureus | Positive | 27 | 16 | 12 | 64 |
| Eastern | Aminoglycosides | Acinetobacter spp. | Negative | 57 | 23 | 9 | 89 |
| Eastern | Carbapenems | Acinetobacter spp. | Negative | 53 | 25 | 2 | 87 |
| Eastern | Fluoroquinolones | Acinetobacter spp. | Negative | 66 | 23 | 15 | 96 |

Table S6b. Summary Statistics for Resistance by Antibiotic Class and Bacteria in Northern Europe.

AvgRest, SDRest, MinRest, MaxRest stands for Average Resistance, Standard Deviation of Resistance, Minimum Resistance and Maximum Resistance, respectively.

| Region | Class | Bacteria | Gram | AvgRest | SDRest | MinRest | MaxRest |
| --- | --- | --- | --- | --- | --- | --- | --- |
| Northern | Aminopenicillins | Enterococcus faecalis | Positive | 4 | 6 | 0 | 35 |
| Northern | High-level gentamicin | Enterococcus faecalis | Positive | 30 | 12 | 10 | 61 |
| Northern | Vancomycin | Enterococcus faecalis | Positive | 2 | 4 | 0 | 33 |
| Northern | Aminopenicillins | Enterococcus faecium | Positive | 87 | 7 | 73 | 100 |
| Northern | High-level gentamicin | Enterococcus faecium | Positive | 48 | 20 | 7 | 88 |
| Northern | Vancomycin | Enterococcus faecium | Positive | 13 | 15 | 0 | 46 |
| Northern | Aminoglycosides | Escherichia coli | Negative | 8 | 3 | 2 | 15 |
| Northern | Aminopenicillins | Escherichia coli | Negative | 51 | 12 | 28 | 72 |
| Northern | Fluoroquinolones | Escherichia coli | Negative | 16 | 6 | 7 | 44 |
| Northern | Third-generation cephalosporins | Escherichia coli | Negative | 9 | 5 | 2 | 36 |
| Northern | Aminoglycosides | Klebsiella pneumoniae | Negative | 16 | 19 | 0 | 64 |
| Northern | Fluoroquinolones | Klebsiella pneumoniae | Negative | 17 | 17 | 2 | 65 |
| Northern | Third-generation cephalosporins | Klebsiella pneumoniae | Negative | 19 | 20 | 1 | 66 |
| Northern | Aminoglycosides | Pseudomonas aeruginosa | Negative | 7 | 8 | 0 | 38 |
| Northern | Ceftazidime | Pseudomonas aeruginosa | Negative | 9 | 6 | 0 | 36 |
| Northern | Fluoroquinolones | Pseudomonas aeruginosa | Negative | 12 | 8 | 0 | 45 |
| Northern | Meticillin | Staphylococcus aureus | Positive | 8 | 8 | 0 | 33 |
| Northern | Macrolides | Streptococcus pneumoniae | Positive | 10 | 7 | 0 | 27 |
| Northern | Penicillins | Streptococcus pneumoniae | Positive | 3 | 4 | 0 | 19 |
| Northern | Carbapenems | Escherichia coli | Negative | 0 | 0 | 0 | 2 |
| Northern | Carbapenems | Klebsiella pneumoniae | Negative | 0 | 0 | 0 | 3 |
| Northern | Carbapenems | Pseudomonas aeruginosa | Negative | 11 | 8 | 1 | 40 |
| Northern | PiperacillinTazobactam | Pseudomonas aeruginosa | Negative | 10 | 7 | 1 | 36 |
| Northern | Aminoglycosides | Acinetobacter spp. | Negative | 20 | 31 | 0 | 90 |
| Northern | Fluoroquinolones | Acinetobacter spp. | Negative | 23 | 34 | 0 | 93 |
| Northern | Carbapenems | Acinetobacter spp. | Negative | 19 | 32 | 0 | 90 |

Table S6c. Summary Statistics for Resistance by Antibiotic Class and Bacteria in Southern Europe.

AvgRest, SDRest, MinRest, MaxRest stands for Average Resistance, Standard Deviation of Resistance, Minimum Resistance and Maximum Resistance, respectively.

| Region | Class | Bacteria | Gram | AvgRest | SDRest | MinRest | MaxRest |
| --- | --- | --- | --- | --- | --- | --- | --- |
| Southern | Aminopenicillins | Enterococcus faecalis | Positive | 5 | 5 | 0 | 24 |
| Southern | High-level gentamicin | Enterococcus faecalis | Positive | 38 | 9 | 12 | 61 |
| Southern | Vancomycin | Enterococcus faecalis | Positive | 1 | 2 | 0 | 7 |
| Southern | Aminopenicillins | Enterococcus faecium | Positive | 87 | 8 | 60 | 98 |
| Southern | High-level gentamicin | Enterococcus faecium | Positive | 46 | 15 | 15 | 66 |
| Southern | Vancomycin | Enterococcus faecium | Positive | 10 | 10 | 0 | 31 |
| Southern | Aminoglycosides | Escherichia coli | Negative | 14 | 4 | 5 | 22 |
| Southern | Aminopenicillins | Escherichia coli | Negative | 59 | 5 | 48 | 68 |
| Southern | Carbapenems | Escherichia coli | Negative | 0 | 0 | 0 | 2 |
| Southern | Fluoroquinolones | Escherichia coli | Negative | 30 | 8 | 15 | 45 |
| Southern | Third-generation cephalosporins | Escherichia coli | Negative | 15 | 6 | 4 | 30 |
| Southern | Aminoglycosides | Klebsiella pneumoniae | Negative | 31 | 14 | 9 | 68 |
| Southern | Carbapenems | Klebsiella pneumoniae | Negative | 13 | 21 | 0 | 68 |
| Southern | Fluoroquinolones | Klebsiella pneumoniae | Negative | 39 | 16 | 14 | 72 |
| Southern | Third-generation cephalosporins | Klebsiella pneumoniae | Negative | 41 | 17 | 10 | 76 |
| Southern | Aminoglycosides | Pseudomonas aeruginosa | Negative | 21 | 10 | 6 | 48 |
| Southern | Carbapenems | Pseudomonas aeruginosa | Negative | 26 | 10 | 13 | 54 |
| Southern | Ceftazidime | Pseudomonas aeruginosa | Negative | 17 | 7 | 5 | 40 |
| Southern | Fluoroquinolones | Pseudomonas aeruginosa | Negative | 27 | 8 | 9 | 48 |
| Southern | PiperacillinTazobactam | Pseudomonas aeruginosa | Negative | 22 | 8 | 6 | 39 |
| Southern | Meticillin | Staphylococcus aureus | Positive | 32 | 11 | 7 | 55 |
| Southern | Macrolides | Streptococcus pneumoniae | Positive | 22 | 6 | 10 | 36 |
| Southern | Penicillins | Streptococcus pneumoniae | Positive | 10 | 9 | 0 | 30 |
| Southern | Aminoglycosides | Acinetobacter spp. | Negative | 64 | 21 | 16 | 92 |
| Southern | Carbapenems | Acinetobacter spp. | Negative | 73 | 22 | 24 | 96 |
| Southern | Fluoroquinolones | Acinetobacter spp. | Negative | 73 | 20 | 28 | 98 |

Table S6d. Summary Statistics for Resistance by Antibiotic Class and Bacteria in Western Europe.

AvgRest, SDRest, MinRest, MaxRest stands for Average Resistance, Standard Deviation of Resistance, Minimum Resistance and Maximum Resistance, respectively.

| Region | Class | Bacteria | Gram | AvgRest | SDRest | MinRest | MaxRest |
| --- | --- | --- | --- | --- | --- | --- | --- |
| Western | Aminopenicillins | Enterococcus faecalis | Positive | 1 | 2 | 0 | 10 |
| Western | High-level gentamicin | Enterococcus faecalis | Positive | 26 | 9 | 7 | 47 |
| Western | Vancomycin | Enterococcus faecalis | Positive | 0 | 0 | 0 | 1 |
| Western | Aminopenicillins | Enterococcus faecium | Positive | 86 | 8 | 61 | 100 |
| Western | High-level gentamicin | Enterococcus faecium | Positive | 39 | 16 | 13 | 73 |
| Western | Vancomycin | Enterococcus faecium | Positive | 5 | 5 | 0 | 24 |
| Western | Aminoglycosides | Escherichia coli | Negative | 7 | 1 | 4 | 10 |
| Western | Aminopenicillins | Escherichia coli | Negative | 53 | 4 | 46 | 63 |
| Western | Carbapenems | Escherichia coli | Negative | 0 | 0 | 0 | 0 |
| Western | Fluoroquinolones | Escherichia coli | Negative | 20 | 4 | 13 | 29 |
| Western | Third-generation cephalosporins | Escherichia coli | Negative | 9 | 2 | 4 | 14 |
| Western | Aminoglycosides | Klebsiella pneumoniae | Negative | 12 | 8 | 2 | 29 |
| Western | Carbapenems | Klebsiella pneumoniae | Negative | 0 | 0 | 0 | 2 |
| Western | Fluoroquinolones | Klebsiella pneumoniae | Negative | 17 | 8 | 5 | 36 |
| Western | Third-generation cephalosporins | Klebsiella pneumoniae | Negative | 16 | 8 | 5 | 36 |
| Western | Aminoglycosides | Pseudomonas aeruginosa | Negative | 9 | 5 | 2 | 24 |
| Western | Carbapenems | Pseudomonas aeruginosa | Negative | 11 | 5 | 3 | 20 |
| Western | Ceftazidime | Pseudomonas aeruginosa | Negative | 9 | 3 | 0 | 17 |
| Western | Fluoroquinolones | Pseudomonas aeruginosa | Negative | 15 | 5 | 6 | 27 |
| Western | PiperacillinTazobactam | Pseudomonas aeruginosa | Negative | 13 | 5 | 3 | 23 |
| Western | Meticillin | Staphylococcus aureus | Positive | 12 | 6 | 1 | 24 |
| Western | Macrolides | Streptococcus pneumoniae | Positive | 14 | 8 | 0 | 31 |
| Western | Penicillins | Streptococcus pneumoniae | Positive | 1 | 2 | 0 | 11 |
| Western | Aminoglycosides | Acinetobacter spp. | Negative | 7 | 4 | 0 | 16 |
| Western | Carbapenems | Acinetobacter spp. | Negative | 5 | 3 | 0 | 12 |
| Western | Fluoroquinolones | Acinetobacter spp. | Negative | 9 | 5 | 0 | 22 |
